# Perforated acute appendicitis resulting from appendiceal villous adenoma presenting with small bowel obstruction: a case report

**DOI:** 10.1186/1471-230X-11-35

**Published:** 2011-04-09

**Authors:** Yu-Guang Chen, Hao-Ming Chang, Yen-Lin Chen, Yi-Chiao Cheng, Chin-Hui Hsu

**Affiliations:** 1 Department of Internal Medicine, Tri-Service General Hospital, National Defense Medical Center, Taiwan; 2 Division of general surgery, Department of Surgery, Tri-Service General Hospital, National Defense Medical Center, Taiwan; 3 Department of Radiology, Tri-Service General Hospital, National Defense Medical Center, Taiwan; 4 Division of gastroenterology, Department of Internal Medicine, Tri-Service General Hospital, National Defense Medical Center, Taiwan

## Abstract

**Background:**

A villous adenoma is an extremely rare benign tumour in the appendix, in contrast to other benign appendiceal lesions. The clinical features are usually asymptomatic. Acute appendicitis is the most common complication with the lesion obstructing the orifice of the appendiceal lumen. Thus, a villous adenoma is usually found during surgical intervention for acute appendicitis. Mechanical obstruction induced by acute perforated appendicitis has been previously reported. Acute appendicitis caused by a villous adenoma presenting with acute intestinal obstruction has not been previously reported.

**Case presentation:**

A 78-year-old woman presented to our Emergency Department with diffuse abdominal pain and tenderness. The abdominal plain film and computed tomography revealed an intestinal obstruction. After surgical intervention, the ruptured appendix was shown to be associated with intestinal obstruction. The post-operative pathologic diagnosis was an appendiceal villous adenoma.

**Conclusions:**

This is the first report describing an appendiceal villous adenoma, which is an occasional cause of perforated acute appendicitis, presenting as a complete intestinal obstruction. We emphasize that in elderly patients without a surgical history, the occult cause of complete intestinal obstruction must be determined. If an appendiceal tumour is diagnosed, an intra-operative frozen section is suggested prior to selecting a suitable method of surgical intervention.

## Background

Intestinal obstruction is a common problem in clinical practice. The common causes of intestinal obstruction are mechanical obstruction or ileus. Although post-operative adhesions account for up to 75% of small bowel obstructions, it is notable that acute appendicitis is a rare cause of small bowel obstruction [1,2]. Acute appendicitis results from obstruction of the appendiceal lumen; the most common causes of obstruction are fecaliths and lymphoid follicular hyperplasia. In contrast, acute appendicitis induced by obstruction with a benign tumour is very uncommon. In particular, villous adenomas are extremely rare appendiceal neoplasms. Early recognition of intestinal obstruction expedites a correct diagnosis.

Clearly, appropriate surgical intervention is very important. Herein we present a case of complete small intestinal obstruction caused by acute appendicitis with perforation resulting from obstruction by a villous adenoma.

## Case presentation

**Figure 1:**
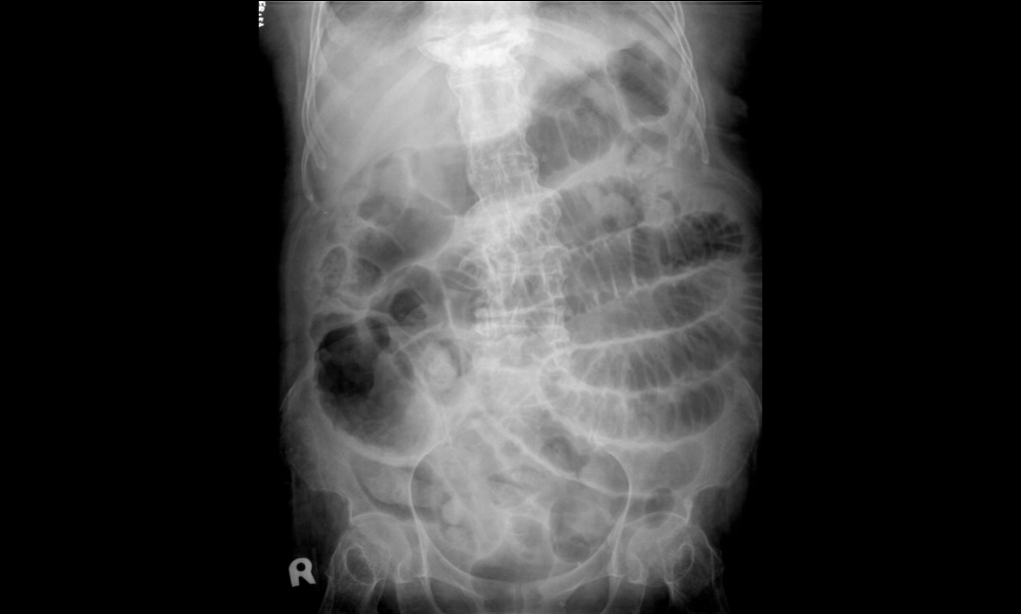
**Abdominal roentography taken during the admission screen revealed dilated bowel. loops with air accumulation in the whole abdomen**.

**Figure 2:**
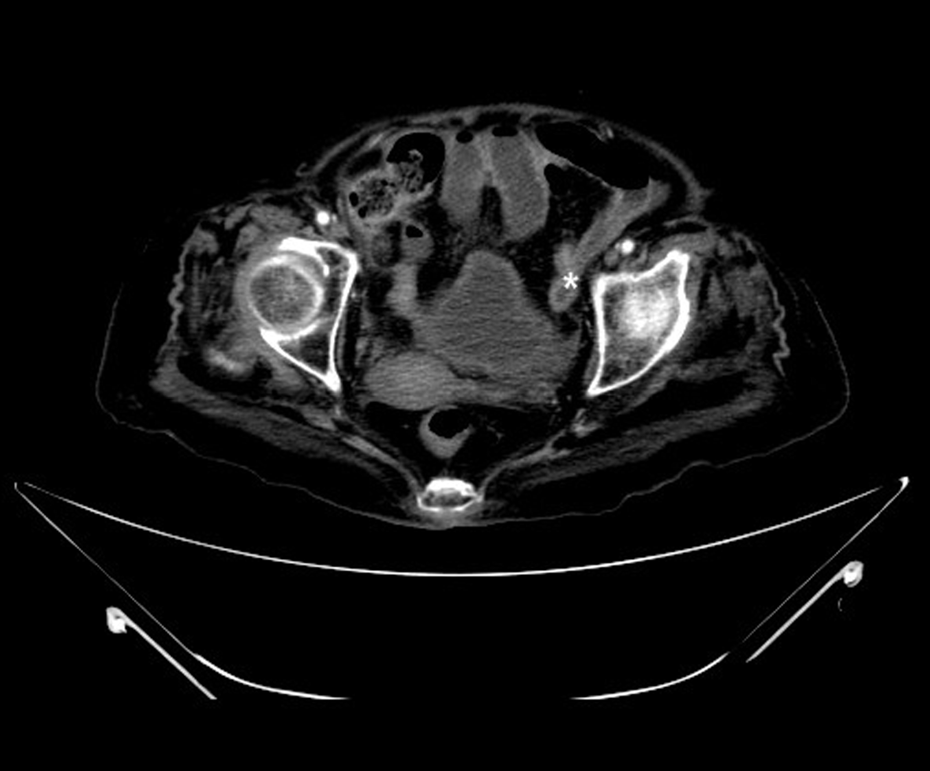
**Abdominal computed tomography**. Coronal view reveals a transitional region (asterisk) at the left lower abdomen.

**Figure 3:**
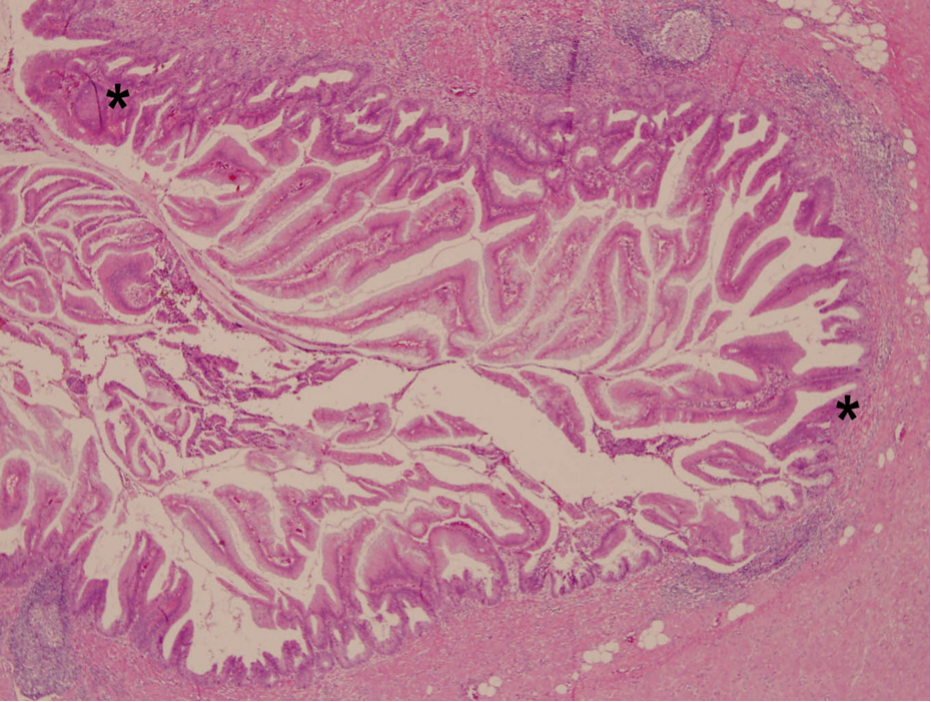
**The appendix tissue reveals villous adenoma with moderate to severe dysplasia (asterisk) located suppurative appendicitis**. (Hematoxylin and eosin stain, original magnification, × 400).

A 78-year-old woman sought evaluation in our Emergency Department with several hours history of diffuse abdominal pain and distension. On physical examination, the patient's blood pressure was 140/70 mmHg, oral temperature was 37.1°C, pulse rate was 108/min, and her respiratory rate was 22 breaths/min. The abdominal examination revealed distension with tympanic percussion and local tenderness, especially over the left lower abdominal region. No obvious muscle guarding or rebound tenderness was noted. Laboratory testing revealed a white blood cell count of 15.5 × 10^3^/μL (95% neutrophils) and a C-reactive protein (CRP) level of 35.20 mg/dL (reference range, <0.5 mg/dL). An abdomen plain film showed dilated bowel loops in the left abdomen (Figure 1). An abdominal computed tomography (CT) scan revealed small bowel distension with an air-fluid level and a transitional region in the left lower abdomen (Figures 2). The patient was treated surgically with an exploratory laparotomy. During surgery, a ruptured inflamed appendix and an adhesion band were found. The histopathological report on the surgical specimen indicated acute suppurative appendicitis and rupture with a villous adenoma that was confined to the mucosa of the appendix and did not extend to the resection margins (Figure 3). The patient was discharged on the 10th post-operative day in a stable condition.

## Discussion

Small bowel obstruction is a common clinical problem. The most common causes of small bowel obstruction is mechanical obstruction, such as adhesions and hernias, which account for >80% of all cases. The clinical presentations of small bowel obstruction include abdominal pain, vomiting, constipation, and fever. The physical examination shows abdominal distention, tenderness, or guarding. Acute appendicitis is known as a cause of small bowel obstruction, which in such cases may be mechanical or the result of an ileus. The obstruction may result from adhesions caused by peri-appendicular inflammation or an ileus because of generalized or localized peritonitis caused by a perforated acute appendicitis. When the two diseases co-exist, the clinical manifestations of the intestinal obstruction usually dominate the clinical symptoms and mask the usual presentation of appendicitis [2].

Luminal obstruction is the pathologic hallmark of acute appendicitis. The most common cause of luminal obstruction is fecaliths, followed by enlarged lymph nodes and tumours. Tumours of the appendix are very uncommon and account for approximately 1% of appendectomies [3]. These lesions are usually diagnosed at the time of surgery or autopsy. Based on previous classic studies involving appendectomy specimens, most appendiceal tumours are benign and consist of a cystadenoma and a villous adenoma [3-5].

Reports of villous adenomas are extremely rare. In general, villous adenomas are neoplasms that most often occur in the rectum and sigmoid colon [6]. Most villous adenomas are found incidentally during histopathological examinations after appendectomy. The frequency of primary appendiceal villous adenomas is approximately 0.06% in appendectomy specimens [7]. Although benign neoplastic lesions located in the appendix are extremely rare, when detected, acute appendicitis is the most common clinical presentation [8].

The pathophysiology of acute appendicitis caused by appendiceal benign tumours is considered to be obstruction in the lumen. Cystadenomas can secrete mucin followed by a mucus-filled appendix. Rupture of the appendix can lead to clinical features of pseudomyxoma peritonei (PMP) complicated with intestinal obstruction [9]. Villous adenomas have a similar clinical picture as cystadenomas [10]. The computed tomography (CT) characteristics of PMP include multiple complex cystic masses of fat density in the peritoneum, omental thickening, and compression of varying degrees of visceral organs and structures. However, no obvious findings on imaging involving villous adenomas with PMP were noted in our case. Thus, the intestinal obstruction was caused by direct rupture of the appendix without PMP. The site of adhesions located over the left lower quadrant near the pelvic region was probably induced by intra-abdominal pus formation.

In our patient, no previous presentations of acute appendicitis, such as local tenderness, local rigidity over the right lower quadrant abdomen, or McBurney's point tenderness were found on the initial examination, for the following reasons.

First, the peritoneum contained the perforated appendix within the sac, preventing widespread inflammation. Thus, in this patient the presentation was limited to local tenderness, and no muscle guarding or peritoneal signs were noted. Second, the co-existence of the two diseases might change the typical pathogenesis of this condition. In general, the sudden onset of complete small bowel obstruction with adhesion band formation in patients without a surgical history must be carefully investigated to identify the underlying problems of this simple disease.

The current opinion regarding the exclusion of malignant lesions depends on whether or not the resection line of the appendiceal stump is clear. Appendiceal villous adenomas have a propensity to develop into invasive adenocarcinomas, such as adenomas, elsewhere in the colon. Thus, an intra-operative frozen section is suggested. Appendectomy is the treatment of choice for those cases in which the lesion does not reach the line of resection [11]. Once margin invasion is found, a right hemi-colectomy should be undertaken. Further therapy and evaluation of an association with neoplasia elsewhere must be considered [12]. Associations between synchronous or metachronous colon cancer (adenocarcinoma) and appendiceal adenomas have been noted. Thus, a colonoscopic examination may play an important role in patients with incidentally-discovered appendiceal tumours, and is particularly indicated in patients in the 6th-8th decades of life [12,13].

## Conclusions

This is the first report of an unusual presentation of perforated appendicitis induced by an intraluminal villous adenoma. No previous report of a similar presentation (complete intestinal obstruction) exists in the literature. We emphasized two key points. First, acute appendicitis and complete intestinal obstruction are surgical emergencies. However, in the elderly with a complete mechanical intestinal obstruction without a previous surgical history or malignancy, the possibility of occult reasons or a co-existing disease, such as acute appendicitis or hernias, must be considered. Second, an appendiceal villous tumour must be considered as a possible cause of acute appendicitis. A post-operative colonoscopy should be considered to check for synchronous or metachronous colon cancer.

## Competing interests

The authors declare that they have no competing interests.

## Authors' contributions

YGC and YLC was involved in writing, reviewing, editing and finalizing the manuscript. HMC and YCC managed the whole operative procedures. YGC, YCC and HMC participated in the care of the patient and assisted in drafting the manuscript. CHH was the specialist in the gastroenterology involved with the case and also reviewed, edited and finalized the manuscript. All authors have read and approved the final version.

## Pre-publication history

The pre-publication history for this paper can be accessed here:

http://www.biomedcentral.com/1471-230X/11/35/prepub
